# Emerging strategies in the sustainable removal of antibiotics using semiconductor-based photocatalysts

**DOI:** 10.3762/bjnano.16.21

**Published:** 2025-02-25

**Authors:** Yunus Ahmed, Keya Rani Dutta, Parul Akhtar, Md Arif Hossen, Md Jahangir Alam, Obaid A Alharbi, Hamad AlMohamadi, Abdul Wahab Mohammad

**Affiliations:** 1 Department of Chemistry, Chittagong University of Engineering and Technology, Chattogram-4349, Bangladesh; 2 Institute of River, Harbor and Environmental Science, Chittagong University of Engineering and Technology, Chattogram-4349, Bangladesh; 3 Department of Civil Engineering, Chittagong University of Engineering and Technology, Chattogram-4349, Bangladesh; 4 Water Management & Treatment Technologies Institute, Sustainability & Environment Sector, King Abdulaziz City for Science and Technology (KACST), Riyadh, 11442, Saudi Arabia; 5 Department of Chemical Engineering, Faculty of Engineering, Islamic University of Madinah, Madinah 42351, Saudi Arabia; 6 Chemical and Water Desalination Engineering Program, College of Engineering, University of Sharjah, Sharjah 27272, United Arab Emirates

**Keywords:** antibiotics, degradation pathways, heterojunctions, mechanisms, photocatalysts, semiconductor

## Abstract

In the constantly growing field of environmental sustainability, the threat of newly discovered pollutants, particularly antibiotics, has become a crucial concern. The widespread presence of these pharmaceutical substances in water sources presents a complex hazard to human health and ecological balance, requiring immediate and novel intervention techniques. Regarding this, semiconductor-based photocatalysts have appeared as promising candidates, providing a sustainable and efficient way to remove antibiotics from aquatic ecosystems. Nanomaterials can effectively and precisely break down and neutralize antibiotic compounds with high efficiency and selectivity by utilizing a complex interaction between radical reactive oxygen species and non-radical equivalents under light irradiation. Although photocatalysts have certain drawbacks, such as a limited capacity to absorb light and concerns about catalytic stability, photocatalysis outperforms other advanced oxidation processes in multiple aspects. This study focuses on summarizing recent advances in the sustainable removal of antibiotics using semiconductor-based photocatalysts. By reviewing the latest studies and sustainable technologies, this study presents new insights into the complex relationship between contaminants and catalytic degradation processes. Compared to single and binary photocatalysts, modified ternary composites were found to have superior photodegradation performance under visible light exposure. To be specific g-C_3_N_4_-based ternary photocatalysts exhibited more than 90% degradation of tetracycline and sulfamethazine antibiotics within one hour of irradiation. This study addresses the antibiotic degradation efficiency during photocatalytic processes and suggests new approaches to improve the performance and scalability for wider use in real-world situations.

## Introduction

Antibiotics are chemical substances used to treat bacterial infections in humans, animals, aquaculture, and livestock feed [1]. Their global use has increased significantly, reaching an estimated 100,000 to 200,000 tonnes between 2010 and 2019 [2], with approximately half designated for animal feed, projected to escalate to 105,596 tonnes by 2030 [3–4]. The widespread and often excessive use of antibiotics has raised public concern, especially because they are environmental contaminants originating from human and animal waste [5]. These antibiotics can persist without change or as active metabolites in the environment, posing significant toxicity risks to aquatic and human life [6–8]. The continuous presence of antibiotics in natural environments can contribute to the development of antibiotic-resistant bacteria (ARBs) and their resistance genes (ARGs), hastening the spread of antibiotic resistance [9–10]. Several studies have reported that this situation poses significant risks to human health and ecological systems [11–16]. Alarming projections in the USA anticipated that antimicrobial resistance-related deaths exceeding the combined toll of cancer and diabetes, with approximately 23,000 deaths annually [17].

**Figure 1:**
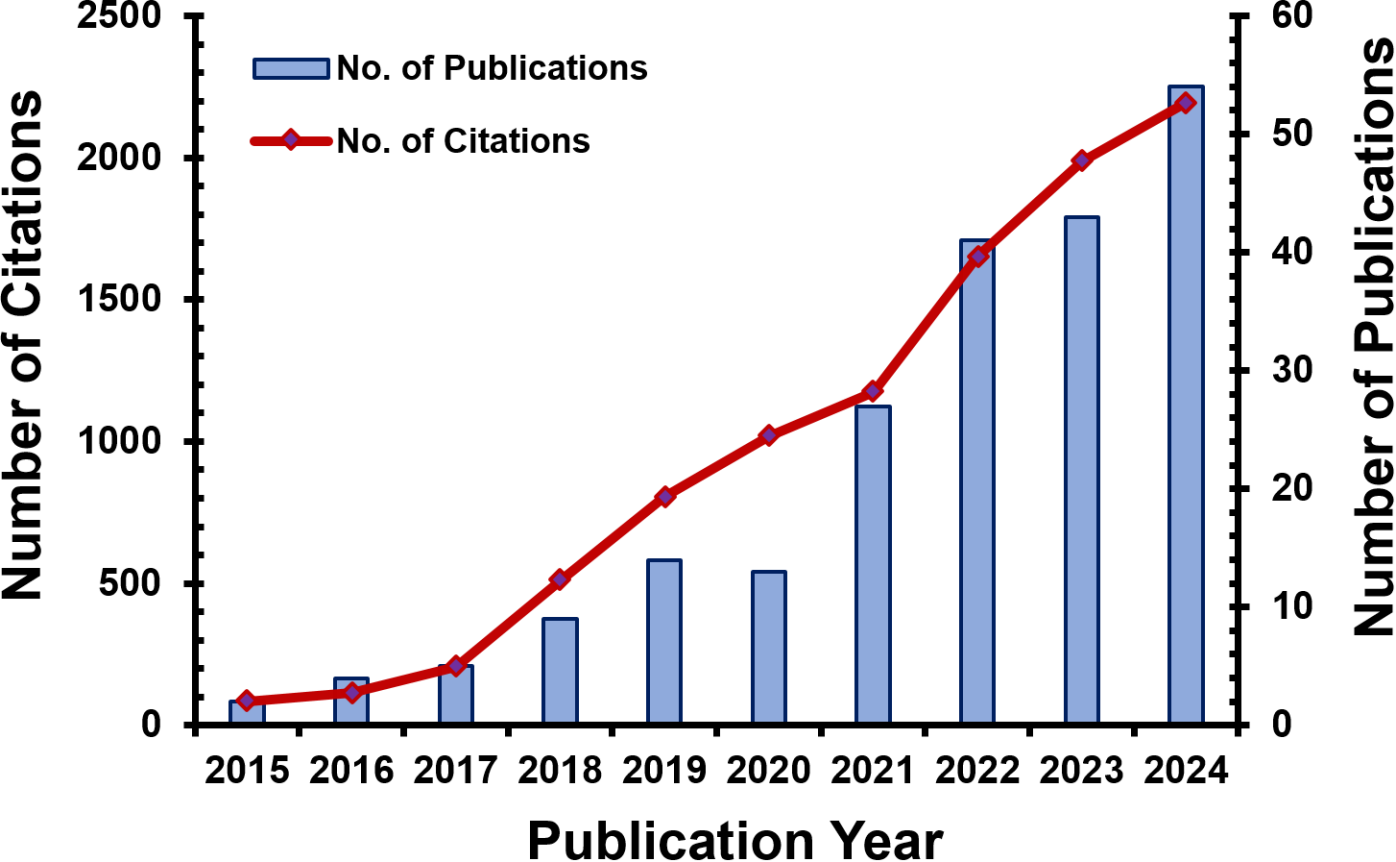
Publications and citations regarding antibiotic degradation using semiconductor-based photocatalysts (Scopus database).

For the mitigation of environmental hazards caused by antibiotics, several physical, biological, and chemical methods have been applied [13,18–19]. Various physical wastewater treatment techniques are based on mechanical separation to reduce contaminant levels by relocating rather than degrading antibiotics [7–8,20]. Biological approaches, such as the activated sludge process and biological membrane technologies, are commonly applied in wastewater treatment [21]. However, these technologies have come under scrutiny because of certain limitations, such as extended processing times and the generation of heat [22]. Conversely, chemical methods such as degradation and solid–liquid separation are also commonly used in making the pollutants non-toxic or non-hazardous [13,23]. It should be noted that many of the conventional methods fail in degrading antibiotics completely since most antibiotics are very complex in structure and are even resistant to biodegradation in an aquatic medium [24]. As a result, antibiotics have been found in different water sources from rivers to lakes, streams, and groundwater sources in many regions. Advanced oxidation processes (AOPs) have lately arisen as very effective treatment technology that has proven to remove antibiotics more effectively than the previous methods of physical adsorption, flocculation, and chemical oxidation [7–8,25]. AOPs offer several advantages, including simple equipment, straightforward operation, minimal sludge production, and the rapid generation of mineralized products. Additionally, they are highly effective in degrading high-strength organic and refractory chemicals [26]. Photocatalyst-based AOPs represent a promising strategy for eliminating antibiotics from polluted water, providing several advantages over other oxidation techniques. By utilizing light energy to activate catalysts, these AOPs facilitate antibiotic degradation without extra chemical oxidants and with minimal harmful byproducts, promoting environmental sustainability [27]. Moreover, they exhibit high selectivity towards antibiotics while preserving water quality. Compared to other photocatalysts material, semiconductor-based photocatalysts often achieved superior efficiency and high mineralization rates, offering a comprehensive solution for antibiotic contamination (see below in Tables 1–6). The progressively increasing trend of publications and corresponding citations in recent times highlight the superiority of semiconductor-based photocatalysts for the degradation of antibiotics (Figure 1). The appeal of photocatalysis lies in its potential to achieve extensive mineralization, converting organic pollutants into harmless mineral compounds. Furthermore, its nonselective nature enables it to address a broad spectrum of contaminants, making it a versatile option for environmental remediation across various water and air treatment applications. These features collectively make photocatalysis an attractive approach for addressing pollution challenges in diverse settings.

In recent years, several significant review papers have focused on removing antibiotics through AOPs [28–30]. Articles explored specific AOP methods tailored for antibiotic remediation, such as H_2_O_2_-based AOP [29], Fenton-based AOPs [31], UV-based AOPs [32], UV/chlor(am)ine-based AOP [33], electrochemical-based AOP (EAOP) [34], persulfate and peroxymonosulfate-based AOPs [30] as well as catalytic degradation processes [35], graphene-based materials [36–37], and adsorption processes [7–8]. However, these papers primarily discuss the oxidizing agents or AOP processes and their efficiency in removing antibiotics without focusing on the detailed mechanisms of how these materials or processes degrade antibiotics. They do not address the entry of antibiotics into the environment, their adverse impact on human health and the environment, or the effects of each treatment process in relation to established industrial applications. As a result, there is a lack of understanding about these processes and their practical limitations for large-scale commercial wastewater treatment.

This review concentrates on semiconductor-based photocatalytic processes and their effectiveness in eliminating antibiotics while also addressing their practical constraints. The scope includes discussions on antibiotic sources, pollution risks and impacts, removal challenges, and influential factors. This study aims to provide readers with a comprehensive understanding of the principles and mechanisms underlying semiconductor-based photocatalysts and their modification for enhanced antibiotic degradation from contaminated water. The discussion also includes concluding remarks and future directions of emerging techniques for sustainable wastewater treatment.

## Review

### Sources of antibiotics

**Figure 2:**
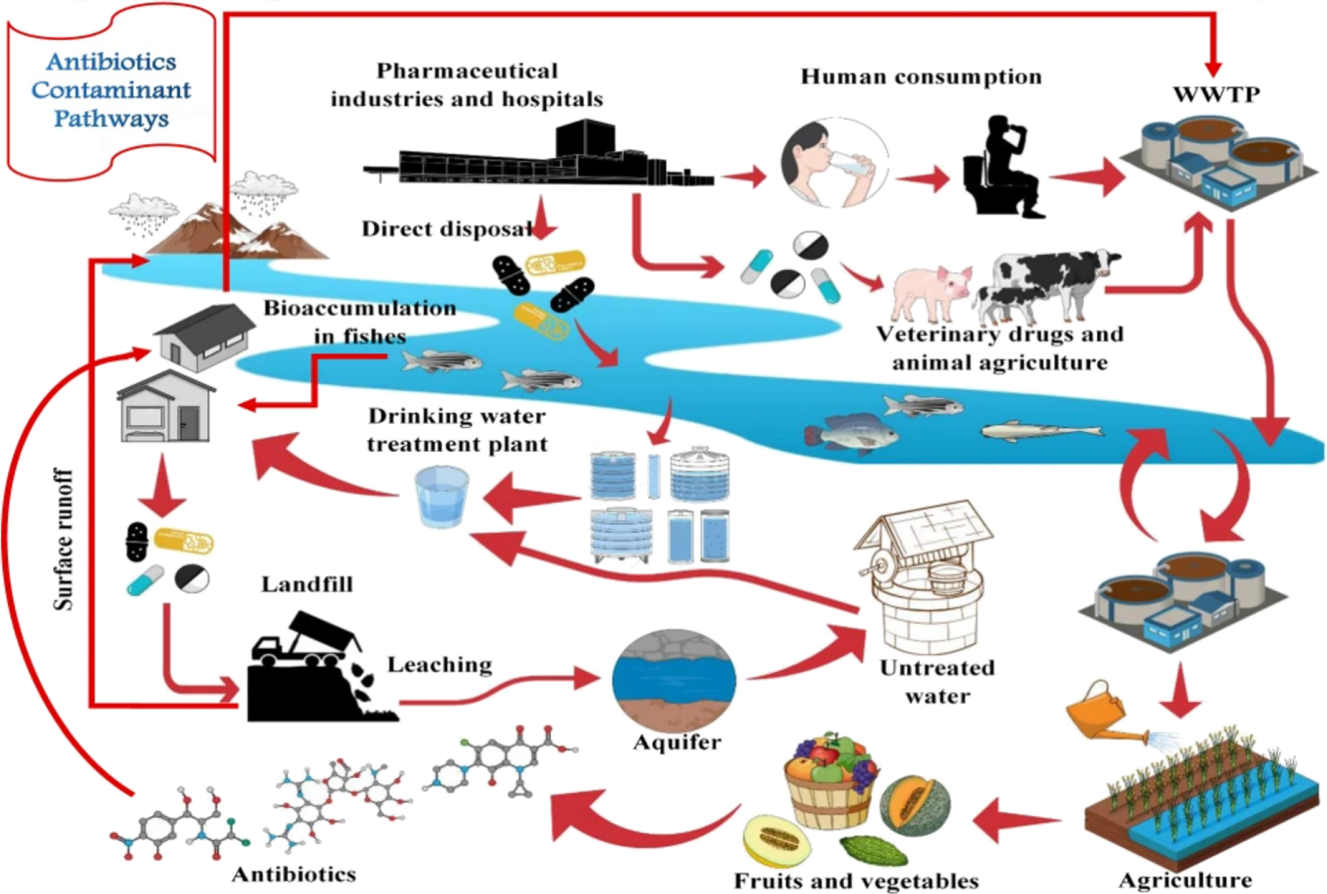
Sources of antibiotics in the environment and their pathways for impacts of antibiotic residues on the environment and human health. Reproduced from [46] (© 2020 C. Bhagat et al., published by Springer Nature, distributed under the terms of the Creative Commons Attribution 4.0 International License, https://creativecommons.org/licenses/by/4.0).

The primary sources of antibiotics in surface water include animal husbandry and aquaculture, domestic sewage discharges, pharmaceutical manufacturing, and healthcare facilities [38]. Antibiotics are commonly administered to animals through feed or water, primarily for growth promotion in large-scale animal farming operations and to prevent and treat infectious diseases [39]. Consequently, antibiotic residues are excreted by the animals in their feces, which can enter the environment by applying manure as fertilizer or runoff from animal housing facilities. Additionally, antibiotics are extensively used in aquaculture, where only a fraction (20–30%) of the pharmaceuticals used are absorbed by the animals [40]. Domestic sewage discharge represents a significant source of antibiotics in urban water systems [41]. Antibiotics are commonly prescribed to individuals to treat various infections. Yet, research indicates that only a small fraction of antibiotics administered to the body are absorbed and utilized by organisms. The remaining 40–90% of active medications or metabolites are excreted in feces and urine [42]. Consequently, treated wastewater or septic effluent containing residual antibiotics can be released into nearby water bodies [43]. Pharmaceutical companies generate, handle, and dispose of significant quantities of antibiotics during production. A study found that downstream water sources from pharmaceutical production plants had significantly higher antibiotic concentrations than upstream sources [44]. Hospitals and healthcare facilities also significantly contribute antibiotics to urban water systems [45]. These facilities administer large doses of antibiotics to patients, resulting in the excretion of antibiotic residues through wastewater. Medical waste incineration is a significant source of antibiotic emissions, potentially contributing to the spread of antibiotic resistance. The present study investigates several primary sources of environmental antibiotics, as illustrated in Figure 2. It provides a comprehensive analysis of their pathways and the mechanisms influencing their movement and persistence in environmental systems.

### Challenges of removing antibiotics from the environment

The challenge of removing antibiotics from the environment, including water bodies, soil, and wastewater, is commonly called the antibiotic removal problem. Antibiotics present in the environment pose risks to both ecosystems and human health. Several primary barriers contribute to the difficulty in removing antibiotics effectively:

#### Chemical complexity

The diverse chemical structures of antibiotics present a significant challenge for their removal compared to simpler contaminants. Various classes of antibiotics necessitate specific treatment processes to achieve effective removal. Furthermore, the degradation of antibiotics can result in the formation of transformation products [47], which may possess distinct properties and introduce additional environmental and health risks.

#### Low quantities

Antibiotics are frequently present in the environment in small amounts, usually in the range of parts per billion (ppb) or parts per trillion (ppt) range. Detecting and eliminating them at such low concentrations poses a significant challenge [42]. Additionally, antibiotics may be co-present with a mixture of other pollutants in certain instances, complicating the isolation and targeted removal of antibiotics.

#### Multiple sources

Antibiotics can enter the environment through various pathways, including wastewater discharges from healthcare facilities, pharmaceutical industry effluents, agricultural runoff, and human and animal waste [48]. However, effectively locating and regulating each source poses a significant challenge.

#### Persistence nature

Certain antibiotics exhibit persistence and resist environmental degradation, resulting in prolonged contamination [49]. Additionally, they are classified as emerging contaminants because of ongoing research on their potential hazards and environmental impacts [50]. Consequently, standardized removal methods may not be readily available.

#### Cost and infrastructure

Implementing successful antibiotic removal technologies often requires substantial investments in advanced treatment infrastructure at high operational cost. While advanced treatment processes like advanced oxidation or membrane filtration may be effective, they are energy-intensive and expensive to implement on a large scale [28].

#### Limited public awareness

Many people may be unaware of how improper antibiotic disposal harms the environment or of the importance of antibiotic removal, which leads to poor practices and pollution. Additionally, in many areas, there may be a lack of regulatory regulations for safe antibiotic levels in water [51], making it challenging to establish treatment targets.

### Principal mechanisms of photocatalytic processes for antibiotic degradation

Semiconductor-based photocatalysis effectively promotes the degradation of antibiotics from contaminated water. Researchers have conducted experiments to evaluate the efficacy of various photocatalysts in eliminating different antibiotics from their respective environments. Research on catalyst composition and application has evolved through four distinct generations [52], each shedding light on the mechanisms of various photocatalysts used for pollutant degradation, namely, single-component transition metal oxides (TMOs) representing the first generation, doped TMOs, binary TMOs, and doped binary TMOs as the second generation, and inactive/active support-immobilized TMOs as the third generation, while the fourth generation refers to ternary/quaternary compositions. The first two generations represent suspended catalysts, the third generation is supported catalysts, and the fourth generation can be suspended or supported. Generally, oxides of titanium, zinc, bismuth, and tungsten, as well as graphene, graphitic carbon nitride (g-C_3_N_4_), and their substitute materials are commonly synthesized and used as photocatalysts for the removal of antibiotics from contaminated sources. These materials are synthesized through a variety of advanced methods, including sol–gel, hydrothermal, solvothermal, precipitation and template-assisted techniques [53]. The synthesis method chosen often depends on factors such as the desired crystal structure, particle size, surface area, and photocatalytic activity required for the specific application.

#### General mechanisms of the photocatalysis process

**Figure 3:**
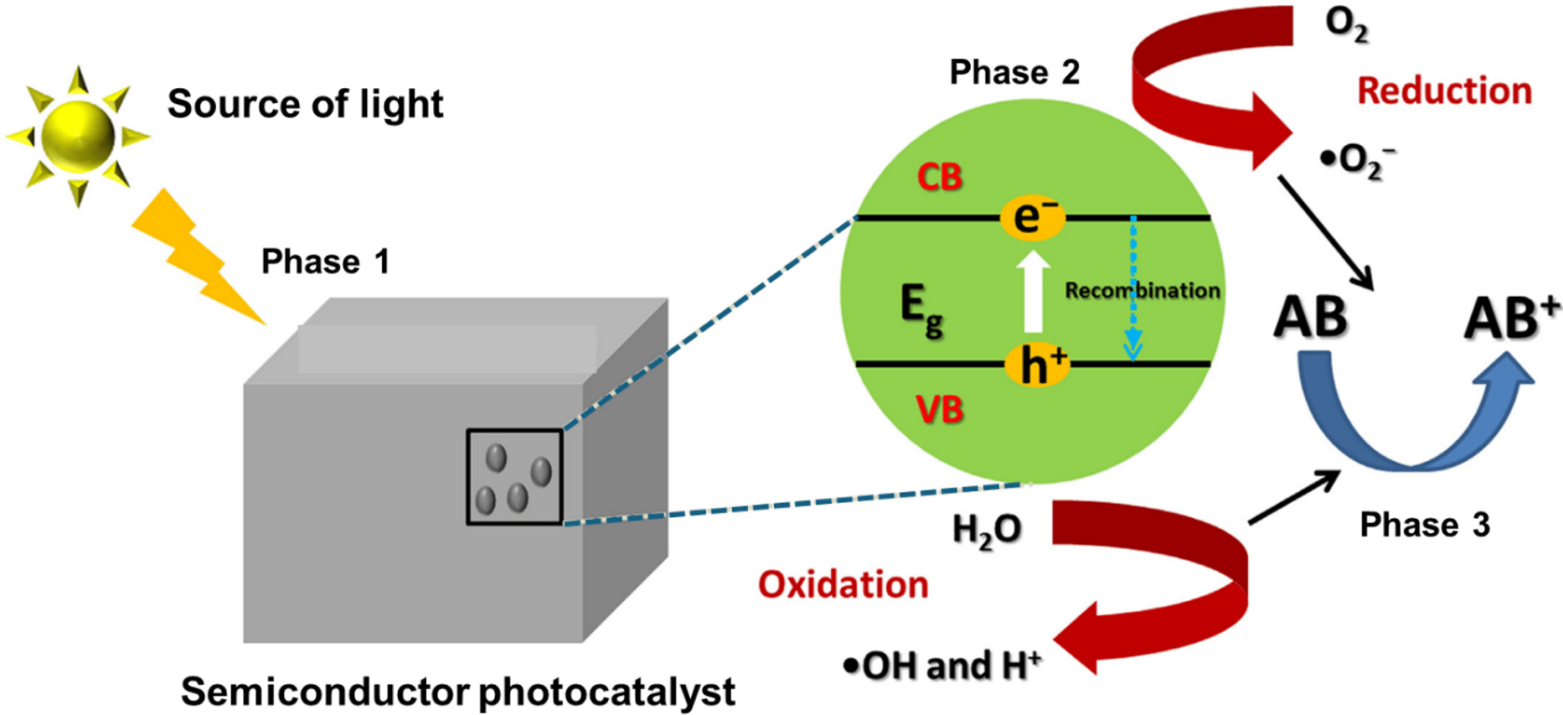
General mechanism for antibiotic degradation using semiconductor-based photocatalysis processes. Reproduced from [35] (© 2022 X. Bai et al., published by MDPI, distributed under the terms of the Creative Commons Attribution 4.0 International License, https://creativecommons.org/licenses/by/4.0).

Three fundamental steps can be identified in semiconductor photocatalysis for the breakdown of antibiotics in contaminated water, that is, photon absorption, excitation, and reaction [54–55]. When a photocatalyst absorbs photons with energy higher than its bandgap, electrons (e^−^) in the valence band (VB) transition to the conduction band (CB), resulting in the formation of holes (h^+^) in the VB (photocatalyst + hν → photocatalyst + h^+^ + e^−^) [54–55]. Afterwards, the electrons and holes are effectively separated and move toward the surface of the photocatalyst, initiating further reactions on the material surface. Photogenerated holes have the potential to directly target antibiotics (h^+^ + antibiotics → H_2_O + CO_2_ + degradation products). This process can possibly result in substantial destruction of the harmful antibiotics. Figure 3 illustrates the different phases of the typical photocatalytic decomposition of antibiotics.

Materials scientists have suggested and acknowledged two distinct degradation pathways [56]. The first pathway occurs when the semiconductor’s CB potential is more negative than the O_2_/O_2_^•−^ redox potential (−0.13 eV vs normal hydrogen electrode (NHE)). In this reductive pathway, the photoexcited electrons have the ability to interact with electron acceptors, like O_2_, which can be found on the catalyst surface or dissolved in water. This reaction reduces O_2_, forming a superoxide radical anion (O_2_^•−^) (O_2_ + e^−^ → O_2_^•−^) [35,55]. Additionally, H_2_O_2_ can be generated by transferring electrons from the conduction band to the adsorbed O_2_. Because the CB of the catalyst has a higher negative potential than the O_2_/H_2_O_2_ system (+0.682 eV vs NHE), the generated H_2_O_2_ subsequently reacts with electrons generated by light to yield active HO^•^ radicals [35,57]. The second pathway also known as the oxidative pathway, occurs when the holes migrate to the surface of the photocatalyst. As a result, HO^•^ radicals are generated by oxidizing H_2_O/OH^−^, and this generation is influenced by the alkalinity or acidity of the surrounding environment (H_2_O/OH^−^ + h^+^ → HO^•^ + H^+^) [35,57]. After excitation, H^+^ possesses the capacity to interact with electrons, generating thermal energy (H^+^ + e^−^ → energy). This process results in a reduction of the photodegradation efficiency. Notably, the typical redox potential of photocatalysts must exceed that of HO^•^/OH^−^ (+1.99 eV vs RHE) [35,57]. Both ROS (HO^•^ and O_2_^•−^) are highly reactive oxidizing agents in photocatalysis. Under extended exposure to high-energy light, they have the ability to efficiently convert antibiotics and their intermediates into the final mineralization products, such as CO_2_ and H_2_O (antibiotics + HO^•^ and/or O_2_^•−^ → CO_2_ + H_2_O).

#### Mechanisms of metal, nonmetal, or co-doped photocatalysts

**Figure 4:**
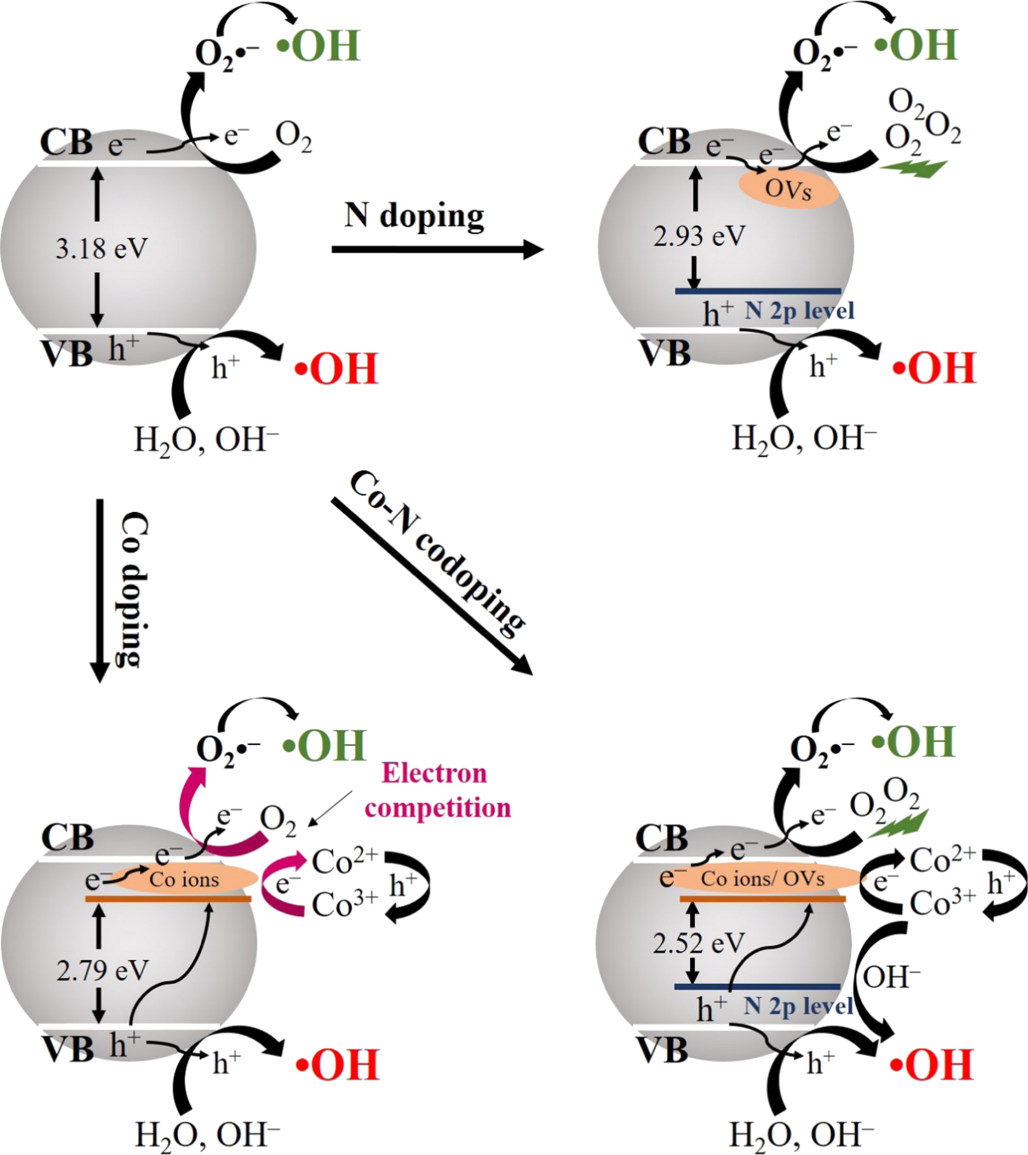
The mechanism of doped ions in semiconductor materials. Figure 4 was reprinted from [60], Journal of Alloys and Compounds, vol. 862, by J.-H. Shen, Y.-H. Tang, Z.-W. Jiang, D.-Q. Liao, J.-J. Horng, “Optimized preparation and characterization of Co-N codoped TiO_2_ with enhanced visible light activity: An insight into effect of dopants on surface redox reactions of photogenerated charge carriers for hydroxyl radical formation”, article no. 158697, Copyright (2021), with permission from Elsevier. This content is not subject to CC BY 4.0.

The large bandgap and high electron–hole recombination rate of traditional and single semiconductor photocatalysts limit their effectiveness under visible light, which hinders their practical application. To improve the photocatalytic efficiency, several dopants, such as transition metals (Fe, Cu, Mn, Zn, Ni, Co, Cr, Ru, and Ag) or nonmetals (C, N, S, and F) have been introduced into the semiconductor material. Metal and nonmetal dopants have the ability to construct an impurity energy level located below the CB of the material [58–59]. This action serves to reduce the bandgap, which in turn extends the absorption wavelength edge towards the region of visible light [60–61]. The idea of modifying semiconductor materials in the second generation involves the process of co-doping with both metal and nonmetal atoms. This method has attracted considerable interest because of its synergistic effect on improving the absorption of visible light and minimizing electron–hole recombination [62–64]. However, these synthesized materials not only remove antibiotic pollutants but also impact the formation of ROS. The co-doped material provide more holes and electrons for the generation of ROS than other monodoped materials. Shen and their group made a comparable observation regarding a Co–N co-doped TiO_2_ photocatalyst, demonstrating that the co-doped TiO_2_ photocatalyst played a significant role in the generation of holes and electrons. Specifically, it contributed 70.1% of the holes and 29.9% of the electrons, in comparison to Co-doped TiO_2_ and N-doped TiO_2_ [60]. This can be attributed to the electron-trapping capabilities of Co ions, which enhance charge transfer and facilitate highly efficient electron–hole separation. The detailed mechanism of dopant ions in semiconductor materials is shown in Figure 4.

#### Mechanisms of heterojunction photocatalysts

**Figure 5:**
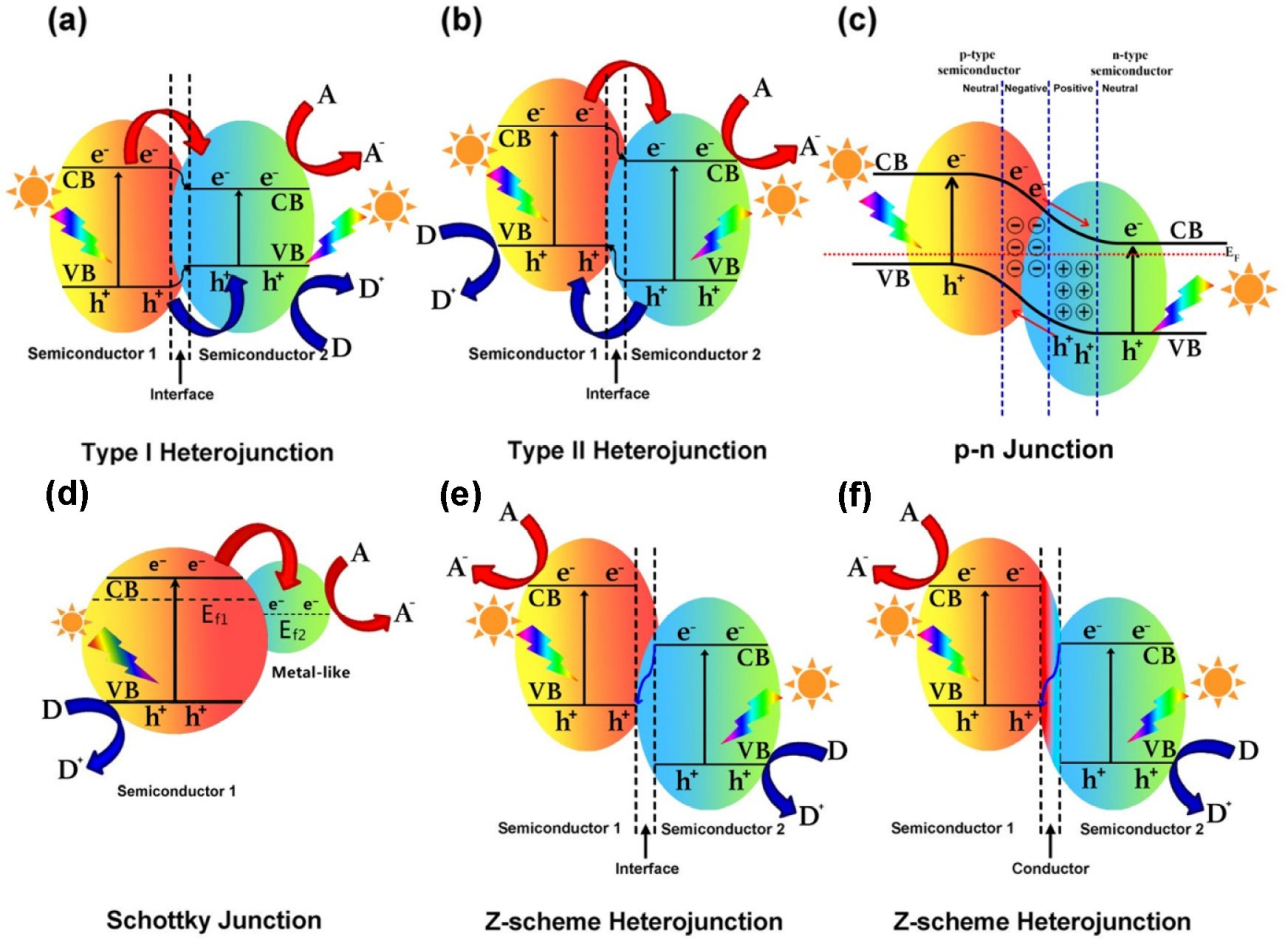
Six structural heterojunction models: (a) type-I heterojunction, (b) type-II heterojunction, (c) p–n junction, (d) Schottky junction, (e) Z-scheme heterojunction (without an electron mediator), and (f) indirect Z-scheme (with an electron mediator). Figure 5 was reprinted from [67], Chinese Journal of Catalysis, vol. 40, by Y. Ren, D. Zeng, W.-J. Ong, “Interfacial engineering of graphitic carbon nitride (g-C_3_N_4_)-based metal sulfide heterojunction photocatalysts for energy conversion: A review”, Pages 289–319, Copyright (2019), with permission from Elsevier. This content is not subject to CC BY 4.0.

Most semiconductor materials are susceptible to electron–hole recombination, which reduces photocatalytic efficacy. As a result, additional efforts must be taken to ensure that visible light is used efficiently while electron–hole recombination is avoided. To improve semiconductor material performance, charge carriers should be separated better, their lifespan extended, the photocatalyst’s bandgap reduced, and the surface area increased [52]. Researchers have taken two approaches to developing effective solar light-activated semiconductor-based photocatalysts. The main approach is to improve the absorption of visible light of semiconductor materials by including metal or nonmetal elements. This augmentation can modify the energy band structure or enable localized surface plasmonic resonance (LSPR). The second strategy focuses on the development of heterojunctions between two semiconductors that is activated by visible light [65–66]. These heterojunctions should have bandgaps and energy levels that match the valence and conduction bands. Heterojunctions can be classified in type-I heterojunctions (Figure 5a), type-II heterojunctions (Figure 5b), p–n junctions (Figure 5c), Schottky junctions (Figure 5d), and Z-scheme heterojunctions (Figure 5e,f), which have all been extensively studied regarding antibiotic photodegradation [67–68]. When a potential difference is applied in heterojunction systems, electrons transfer from the conduction band (CB) of semiconductor 1 (SC1) to the CB of semiconductor 2 (SC2). At the same time, holes in the valence band (VB) of SC1 migrate to the VB of SC2. This charge transfer occurs in type-I heterojunctions, as shown in Figure 5a.

In type-II heterojunctions, however, holes move from SC2 to SC 1 (Figure 5b). In the p–n junction system, a type-II mechanism exchanges electrons and holes. Electrons travel from p-type to n-type semiconductors, whereas holes move from n-type to p-type semiconductors (Figure 5c). The effective segregation of light-induced charge carriers allows the CB of the second semiconductor and the VB of the first semiconductor to engage in reduction and oxidation processes, thus enhancing the photocatalytic activity. Although type-II heterojunctions can restrict photogenerated charge recombination, they also reduce compound semiconductors’ redox capacity and pose problems to the continuous flow of electrons and holes because of electrostatic repulsion. A Schottky junction is also formed by combining two different semiconductor materials (Figure 5d). The oxidation capability of Schottky heterojunctions is restricted to some organic impurities because of the location of the semiconductor photocatalyst’s VB. To overcome the aforementioned concerns with other heterojunctions, the concept of Z-scheme heterojunctions was developed, inspired by natural photosynthesis [69–71]. These heterojunctions are classified based on their charge transfer mechanism and the presence or absence of mediators. The direct Z-scheme relies on the direct electron transfer between photocatalysts; eliminating the electron mediator simplifies the design and enhances stability but may suffer from higher charge recombination. In contrast, an indirect Z-scheme, which uses a redox pair (e.g., Fe^3+^/Fe^2+^) or a conductive mediator to facilitate efficient charge transfer and spatial separation of charge carriers, reduces the risk of recombination and increases the photocatalytic efficiency. While indirect composites are more complicated to fabricate and possibly beset by stability problems due to mediator degradation, indirect Z-schemes are nevertheless often used in applications that demand high charge separation performance. In contrast, direct Z-schemes are best suited for systems where robustness and simplicity are desired. When exposed to visible light, electrons at a lower CB location on SC2 interact with holes on the VB of SC1 via the heterojunction interface (Figure 5e) or an intermediary conducting medium (Figure 5f). This enables the photocatalyst to maintain high redox capacity.

### Efficiency of antibiotic removal by semiconductor-based photocatalysts

Photocatalysis is a highly effective, affordable, and environmentally friendly approach for removing antibiotics from wastewater. Various semiconductor photocatalysts, such as TiO_2_, ZnO, bismuth oxyhalide (BiOX), g-C_3_N_4_, graphene oxide (GO), WO_3_, and their derivatives, have distinct physical and chemical characteristics that influence their efficiency and efficacy in removing antibiotics. The subsequent sections describe these six commonly used semiconductor materials to remove antibiotics from the environment and their impact on sustainable wastewater treatment.

#### Titanium dioxide-based materials

TiO_2_ is the most commonly used photocatalyst for antibiotic removal owing to its unique features. In nanostructured forms, this substance exhibits outstanding physical and chemical durability, a high ratio of surface area to volume, adjustable electronic properties, exceptional photocatalytic performance, lack of toxicity, widespread availability, and cost efficiency [65]. It has a broad bandgap (3.2 eV). Therefore, it can be activated only by UV radiation, which is only a small part of the solar spectrum. This feature makes TiO_2_ not suitable for outdoor applications where natural light is abundant. Although TiO_2_ has a high photocatalytic activity under UV light, its practical use is limited because of rapid electron–hole recombination and insufficient visible light absorption [65]. Hence, it is critical to develop effective strategies to enhance TiO_2_ activity under visible light. When using TiO_2_ in a full-scale field deployment, reducing the amount and utilizing solar energy can be extremely cost-effective and beneficial to wastewater treatment. Researchers have improved the photocatalytic activity of bulk TiO_2_ through various modifications, including doping with a suitable transition metal and synthesizing composite materials. Several advanced photocatalysts have been developed and successfully used to prevent antibiotics contamination, a list of which is shown below in Table 1. These modifications have led to improved charge separation rates, reduced recombination rates, and the generated oxygen vacancies, ultimately enhancing catalytic efficiency in visible light or solar-simulated light. The durability of the modified TiO_2_ relied on the number of electrons in the dopants and their ionic size.

Although researchers have significantly improved the catalytic efficacy of TiO_2_ under visible or solar-simulated light, retrieving the synthesized small particles poses new challenges. Researchers have recently synthesized heterojunctions using TiO_2_ and magnetic particles such as α-Fe_2_O_3_ and Fe_3_O_4_ in order to solve the problem of recovering particles. They accomplished the separation with success, greatly enhancing the efficiency of the catalysts and their potential to be reused, while also decreasing the overall cost of synthesis. The construction of the α-Fe_2_O_3_@TiO_2_ photocatalyst resulted in a higher efficiency in removing the antibiotic cefixime (CFX) than that of pure TiO_2_ and Fe_2_O_3_ under visible light illumination (500 W halogen lamp and wavelengths above 400 nm) [72]. At a dosage of 0.012 g/L of α-Fe_2_O_3_@TiO_2_ and pH 4.76, 98.8% of an initial CFX concentration of 20.5 mg/L was effectively removed after 103 min. The heterojunction formed between TiO_2_ and Fe_2_O_3_ promoted the electron–holes segregation rate and reduced the rate of recombination. A TiO_2_/GO/chitosan photocatalyst was synthesized by Erim et al. [73] for the degradation of CFX under UV-A irradiation. Under optimized conditions (catalyst dose of 0.327 g/L, CFX concentration of 20.29 mg/L, pH 4.1, and UV-A irradiation of 60 W), the TiO_2_/GO/chitosan photocatalyst exhibited a prominent degradation efficiency of 95.34%. They also reported in another article that the SWCNT/ZnO/Fe_3_O_4_ combination exhibited a CFX decomposition efficiency of 94.19% at pH 5.93, 22.76 ppm CFX, and 0.46 g/L photocatalyst [74]. In a recent study, Jeyaprakash and coworkers [75] synthesized Ti^3+^-doped TiO_2_ with oxygen vacancies utilizing ultrasonic treatment for the degradation of tetracycline (TC) via sono-photocatalysis under visible halogen lamp irradiation. The modified TiO_2_ demonstrated remarkable degradation of TC, with an efficiency of approximately 96%, which is 1.56 times higher than that of pure TiO_2_ photocatalysts.

#### Zinc oxide-based materials

Zinc oxide (ZnO) is another widely used semiconductor material that exhibits enhanced sensitivity to ultraviolet (UV) light. It possesses a substantial surface area, exhibits good thermal stability, is non-toxic, and can be easily morphologically modified (such as nanorods, nanosheets, and nanobelts). Because of its higher quantum efficiency, it is anticipated to demonstrate superior photocatalytic activity in comparison with TiO_2_, g-C_3_N_4_, and BiOBr [66]. However, its broader bandgap (3.37 eV) and rapid electron–hole recombination limit its ability to undergo photocatalytic reactions when exposed to visible light [66]. To address these limitations, significant endeavors have been undertaken to improve the properties of ZnO by introducing dopants (nonmetals and/or transition metals compounds) into ZnO. This alteration introduces surface defects, leading to a decrease in the bandgap. As a result, the materials become more efficient in harvesting energy to produce reactive species, which is beneficial for applications involving contaminant treatment [61]. Hunge and coworkers [76] synthesized MoS_2_/ZnO (MZ) composites with a smaller bandgap (2.81 eV), which showed higher ciprofloxacin (CIP) removal efficiency than their single constituents (ZnO = 43% and MoS_2_ = 50%). A similar approach was also observed for the removal of erythromycin (ERY), spiramycin (SP), and CIP by using ZnIn_2_S_4_ [77], g-C_3_N_4_/ZnFe_2_O_4_ [78], Cr_2_O_3_@ZnO [79], and ZnO/TiO_2_ [80]. However, the application of doped ZnO for remediation purposes is presently impractical because of electron–hole recombination under certain conditions.

Scientists have found a variety of adsorbent materials, including biochar, metal-organic frameworks (MOFs), functionalized mesoporous silica, porous organic polymers, zeolite, and derivatives of graphene. These substances act as a support for metal oxides and immobilize the catalyst, increase the catalyst surface area, and improve the chemical stability. Consequently, there is an enhancement in catalytic performance. In a study conducted by Rahman and colleagues [61], it was found that pure ZnO has a very low efficiency (7%) in degrading the antibiotic metronidazole (MNZ) through photocatalysis under 180 min of irradiation with visible light (wavelength, λ ≥ 400 nm and intensity ca. 3 × 10^4^ lux). However, the efficiency increases to 31.8% when using N-doped ZnO photocatalyst. After 70 min of light exposure, a ternary GO-N-ZnO photocatalyst showed a considerable increase in photocatalytic efficiency of 43.45%. Yu and coworkers [81] synthesized ZnO with N,O-containing biochar (ZnO/NOC), which showed a TC removal efficiency of 97.08%. The photocatalytic activities of ZnO/NOC were 5.4 and 7.7 times higher than that of pure ZnO for TC (30 mg/L) under ultraviolet and visible light, respectively. The excellent performance was ascribed to large surface area, proper pore sizes, fast charge transfer, high density and long lifetime of photoinduced charges, and strong interaction between ZnO and N,O-containing biochar [81]. Roy and colleagues [82] effectively synthesized a rGO-ZnO composite functionalized with ferrocene through a simple hydrothermal method. This Fc@rGO-ZnO photocatalyst exhibited exceptional performance compared to pristine ZnO, resulting in a five-fold increase in CIP and SMX removal efficiency. After three hours of treatment, it removed more than 95% of the antibiotics. The most recent research on the use of ZnO- or Zn-based compounds as photocatalysts to remove antibiotics from water is compiled below in Table 2.

#### Bismuth-based materials

Although the activity of titanium dioxide- and zinc oxide-based photocatalysts has been increased through modification, they still absorb visible light poorly. Bi-based photocatalysts have been widely employed to degrade antibiotics under the illumination of UV and visible light [83–84]. They also possess a distinctive electronic structure with the VB containing Bi 6s and O 2p orbitals [54]. This distinctive configuration results in a more pronounced absorption edge in the visible light spectrum. The inclusion of bismuth in photocatalytic research is justified because of its limited solubility in water, which contributes to the compounds’ ecological safety. Nevertheless, Bi-based photocatalysts have certain limitations, including reduced surface area, diminished absorption efficiency, poor heterojunction interface matching, and limited carrier transfer routes [85]. Bi-based materials for the degradation of antibiotics include various compounds such as Sillén-type BiOX, scheelite structures (BiVO_4_, BiOIO_3_, Bi_2_O_3_, and Bi_2_S_3_), Aurivillius-type Bi_2_MO_6_ (M = Mo, Cr, and W), and other composites synthesized with metals and nonmetals or heterojunctions. Table 3 below provides comprehensive details and a concise overview of the latest research on bismuth-based photocatalysts used to eliminate antibiotics from wastewater.

Bi_2_O_3_ is a widely utilized semiconductor in the fields of electronics and chemical engineering because of its uncomplicated composition, affordable price, and easy manufacturing process. Bi_2_O_3_ is widely recognised as a polycrystalline photocatalyst that exhibits four main phases. The majority of these phases are determined to be unstable, with α-Bi_2_O_3_ appearing at low temperatures and δ-Bi_2_O_3_ appearing at high temperatures. Scientists have noted that Bi_2_O_3_ in the metastable phase can undergo a transformation into (BiO)_2_CO_3_, which limits its potential as an effective visible photocatalyst because of its chemical instability [85]. Furthermore, β-Bi_2_O_3_ exhibits enhanced photocatalytic efficiency in comparison to α-Bi_2_O_3_. Nevertheless, the inherent instability of β-Bi_2_O_3_ presents a formidable obstacle in the development of uncomplicated techniques for producing pure β-Bi_2_O_3_, particularly at the nanoscale. The photocatalytic efficiency of Bi_2_O_3_ is inadequate for decomposing antibiotics without modification with suitable dopants. Chen and his colleagues [86] synthesized nanoscale Bi_2_O_3_ and observed little degradation of TC. Modification of Bi_2_O_3_ with MnO_2_, changed the morphology from nanorods to nanosheets. This nanosheet structure showed a higher TC removal efficiency than pure Bi_2_O_3_, attributed to the increased specific surface area and absorption capacity. Using a one-step hydrothermal process, Wu et al. [69] synthesized Bi-bridged Z-scheme BiOCl/Bi_2_O_3_ heterojunctions. The constructed binary photocatalysts exhibited 94.79% TC degradation efficiency owing to the enhanced separation of electron–hole pairs and powerful redox capability. Higher photoactivity was also observed for other heterostructures such as NiFe_2_O_4_/Bi_2_O_3_ [87], Fe_3_O_4_@Bi_2_O_3_–RGO [88], Bi_2_O_3_/(BiO)_2_CO_3_ [89].

Bismuth oxyhalides (BiOX) are renowned as photocatalysts because of their distinctive optical and electrical properties, distinguishing them from other promising materials. The distinctive layered structure of BiOX (X = Cl, Br, and I) facilitates efficient separation of charge carriers, resulting in exceptional photocatalytic performance [85]. BiOX includes [Bi_2_O_2_]^2+^ slabs surrounded by double slabs of [X]^−^. The bandgaps of the different compounds are as follows: 3.22 eV (BiOF), 2.80 eV (BiOCl), 2.36 eV (BiOBr), and 1.75 eV (BiOI) [85]. Although BiOCl has a larger bandgap, it is considered a more promising photocatalyst than BiOI and BiOBr [90]. For example, BiOCl microspheres were able to remove 91.90% of TC after 30 min treatment at a dosage of 500 mg/L of catalyst, at pH 4.8 and an initial TC concentration of 20 mg/L [91]. The degrading effectiveness of the photocatalysts reduced as the amount of sorbitol was increased. The improved photodegradation was possible because the catalyst prevented the recombination of electrons and holes and facilitated the direct attack of h^+^ on the surface of BiOCl [84,91]. This was verified by monitoring the lower effectiveness of BiOI [92] and BiOBr [93] in removing antibiotics. The photocatalytic activity of BiOX varies based on the dipole moment; the efficiency is particularly high when the dipole moment exceeds 2.0 D. The hybridization of halogens does not impact electron mobility. However, it does lead to a decrease in hole mobility while possibly enhancing the separation of charge carriers [94]. In order to enhance the photocatalytic activity of BiOX, significant endeavors have been undertaken, including the creation of heterojunctions or the introduction of metal doping. Wang et al. [84] synthesized BiOCl/Mt photocatalysts in which montmorillonite (Mt), which is naturally rich in iron, was combined with bismuth nitrate. According to the authors, the composite exhibited 3.4 times higher reactivity than BiOCl when exposed to visible light. The degradation of TC using the BiOCl/CdS catalyst exhibited a similar tendency, with degradation rates 4.73 and 2.94 times higher than those of pure CdS and BOCl, respectively [95]. Some other heterostructures also exhibited significant photocatalytic activity in the degradation of antibiotics; they are given below in Table 3.

Bismuth metal oxides such as Bi_2_WO_6_, Bi_2_MO_6_, BiVO_4_, and Bi_2_Ti_2_O_7_ are also gaining attention for their possible use in antibiotic degradation. The hybrid oxides mostly comprise Bi_2_O_3_ and various metal oxides such as V_2_O_5_, W_2_O_3_, Mo_2_O_3_, and TiO_2_. BiVO_4_ is a newly designed n-type semiconductor with enhanced photocatalytic efficiency. This is attributed to its specific physicochemical features, including ferro-elasticity and ionic conductivity [90]. Regrettably, bismuth-based metal oxides exhibit a low light conversion efficiency as a result of the swift recombination of electrons and holes. However, several composite photocatalysts have been studied for the degradation of antibiotics, including BiVO_4_/MoS_2_, BiVO_4_/FeVO_4_, AgI/BiVO_4_, g-C_3_N_4_/BiVO_4_/rGO, Ag/AgBr/BiVO_4_, CuS/BiVO_4_, BiVO_4_**/**TiO_2_/rGO, MnFe_2_O_4_/BiVO_4_, BiVO_4_/GO/CoFe_2_O, ZnFe_2_O_4_/BiVO_4_/g-C_3_N_4_ and BiVO_4_@BiOCl (see below in Table 3). A novel heterostructure of BiVO_4_ nanosheets and MoS_2_ nanoflakes yielded 97.46% degradation of TC within 90 min of visible light illumination with 40 mg/L initial concentration of TC and 15 mg/L catalyst loading [96]. Surprisingly, the photocatalytic TC degradation performance decreased to 66.82% when the catalyst loading was increased to 20 mg/L. The stability of the nanocomposite photocatalyst remained at 94.45% after 4th cycle of reuse. Trapping analysis showed that scavengers reduced the photodegradation rates.

Bismuth tungstate (Bi_2_WO_6_) is also an n-type semiconductor that belongs to the Aurivillius phases. The material exhibits two distinct crystallographic phases, namely monoclinic and orthorhombic. Orthorhombic Bi_2_WO_6_ has alternating layers of (Bi_2_O_2_)^2+^ and WO_6_ octahedrons. It comprises perovskite layers and has a bandgap of 2.77 eV. The research community has shown significant interest in zero-dimensional Bi_2_WO_6_ quantum dots and one-dimensional Bi_2_WO_6_ nanofibers. Bismuth tungstate exhibits excellent thermal and chemical stability in addition to its activity in visible light. It is typically synthesized through mixing Bi(NO_3_)_3_ and Na_2_WO_4_ using different methods. The distinctive layered structure of this material creates gaps between the slabs that form an electric field. This electric field opposes the movement of electrons and holes. This effect decreases the recombination of charge carriers, leading to an increase in photocatalytic activity. In a recent study, Wang et al. [97] found that small amounts of added Ti greatly enhanced the chloramphenicol (CAP) degradation capabilities of Bi_2_WO_6_, resulting in an impressive degradation rate of 92.44%. It has been observed that the incorporation of Mg, Fe, Zn, Cu or other transition metals through doping can potentially improve Bi_2_WO_6_ light absorption capabilities and increase the antibiotic degradation efficiency [98]. Some metal-doped bismuth photocatalysts and their antibiotic degradation efficiency are summarized below in Table 3.

#### Graphitic carbon nitride-based materials

Graphitic carbon nitride (g-C_3_N_4_) is a metal-free semiconductor material with distinct optical, electrical, structural, and physicochemical characteristics [99]. These properties make it well-suited for applications in energy and environmental fields. Because of its narrow bandgap of 2.7 eV, this material can efficiently absorb a significant amount of visible light, which is advantageous for both oxidation and reduction reactions [100]. Nevertheless, some noteworthy concerns arise, such as elevated rate of electron–hole recombination, limited surface area, restricted number of active sites, slow kinetics of surface reactions, and the reduced mobility of charges, resulting in electron delocalization [99–100]. The molecular rearrangement of g-C_3_N_4_ has been the subject of recent research because of its potential to alter the surface chemistry and textural structure [101]. This technique reduces carrier transfer resistance, improves pollutant adsorption, broadens the light absorption range, and promotes carrier separation. During the degradation of tetracycline hydrochloride (TCH), g-C_3_N_4_ exhibited remarkable activity under visible light and degraded 84.1% of TCH, while bulk g-C_3_N_4_ achieved only 52.1% after 90 min of irradiation.

Recently, researchers have revealed that adding small organic compounds into the precursors of g-C_3_N_4_ through copolymerization can significantly improve the efficiency of g-C_3_N_4_. Li et al. [102] carried out the copolymerization of thiourea with 7,7,8,8-tetracyanoquinodimethane (TCNQ) to enhance the energy band and electronic structure of g-C_3_N_4_. The pefloxacin degradation efficiency of the g-C_3_N_4_/TCNQ catalytic system was four times higher than that of pristine g-C_3_N_4_. By combining thiourea with 3-aminopyridine, researchers modified the morphology and textural structure of g-C_3_N_4_, which improved its solar absorption and charge-carrier transportation. The photocatalytic TC decomposition rate was 3.32 times higher in pyridazine-doped g-C_3_N_4_ than in the unmodified form [103].

Several studies have shown that heterojunctions formed by combining g-C_3_N_4_ with other semiconductors with comparable band structures can take advantage of the differences in energy band structures while simultaneously combining the best features of both components. The result is an intrinsic electric field between the materials, which slows down the recombination of photogenerated electrons and holes and speeds up the transfer of photogenerated carriers [104]. By combining ZnO and g-C_3_N_4_, Wang et al. [105] synthesized a Z-type heterostructure. The newly synthesized composite had greater interlayer spacing, specific surface area, and pore volume. After 120 min of simulated light exposure, the rates of TC degradation by pure ZnO, g-C_3_N_4_, and defective ZnO/g-C_3_N_4_ composite were found to be 35.20%, 71.48%, and 93.47%, respectively. Because of the existence of N defects, the constructed nanocomposite promotes the electron transfer efficiently with lower recombination rates. Other g-C_3_N_4_ heterojunction photocatalysts, such as CdS/g-C_3_N_4_ [106], CoO/g-C_3_N_4_ [107], and P-doped g-C_3_N_4_/Co_3_O_4_ [58], have also shown improved photocatalytic activity (see below in Table 4). The binary composite still has several shortcomings. Compared to the binary composite, adding a third semiconductor can improve the separation of charge carriers and expand the range of wavelengths that can be absorbed because of synergistic effects. As an example, Kumar and colleagues synthesized a ternary S-scheme K,P-codoped g-C_3_N_4_/GO/CoFe_2_O_4_ (KPCN/GO/CoFe_2_O_4_) photocatalyst, which demonstrated a greater TC removal efficiency compared to the single constituents and binary heterostructures [64]. The photodegradation efficiencies of TC for KPCN/GO/CoFe_2_O_4_, KPCN/GO, KPCN, and g-C_3_N_4_ were reported to be 85%, 57%, 42%, and 30% after 60 min of visible light exposure, respectively. Likewise, Liu et al. [108] designed a ternary g-C_3_N_4_/Ag_2_CO_3_/GO photocatalyst, which follows a double Z-scheme, to degrade TC. The ternary system exhibits potent oxidation and reduction capabilities for antibiotic degradation (81.6% within 60 min) compared to the binary g-C_3_N_4_/Ag_2_CO_3_ composite (67.5% within 60 min). Indirect Z-scheme photocatalytic systems differ from direct Z-scheme systems by incorporating an electron mediator between the two semiconductors. This mediator facilitates the transport and separation of electrons and holes. However, the inclusion of an ionic electron transport medium in the traditional Z-scheme photocatalytic system leads to the occurrence of reverse reactions on the surface of photocatalysts, resulting in a decrease in the total number of photogenerated charges. Samsudin and colleagues [109] validated their findings by designing a Z-scheme Ag/AgVO_3_/g-C_3_N_4_ photocatalyst. This catalyst exhibited outstanding capabilities in degrading CIP (82.6% within 2 h) and generating hydrogen from rainwater. The effective separation and mobility of photogenerated charge carriers were credited to the role of Ag nanoparticles as electron mediators. There are some other observations, which are shown below in Table 4.

#### Graphene oxide-based materials

Graphene is a monolayer of carbon atoms organized in a hexagonal lattice, with various types of defects present around the edges. This material is categorized based on the level of surface oxidation, which includes pristine graphene, graphene oxide, and reduced graphene oxide (rGO). Among the derivatives of graphene, GO and rGO are frequently used to support photoactive materials and immobilize pollutants. Additionally, they serve as crucial interfaces for electron carriers, augmenting light absorption and antibiotic adsorption through their functional groups [110]. Graphene derivatives possess two notable drawbacks. They are more expensive than other carbon compounds, and their normal synthesis procedure requires using dangerous oxidizing and reducing agents [110]. Therefore, it is crucial to utilize direct, secure, economical, and eco-friendly synthesis methods to use these materials as photocatalysts.

GO has attracted interest as another metal-free carbon material, owing to its two-dimensional ultrathin layered structure, robust stability, and remarkable capacity to transport charge carriers [110]. When coupled with suitable semiconductor materials or modified to other forms of heterostructures, it can enhance the photocatalytic efficiency for various applications, including water purification. GO-based photocatalysts can facilitate this process by generating electron–hole pairs upon absorption of photons. Recently, Kumar et al. [64] fabricated a K,P-co-doped g-C_3_N_4_/CoFe_2_O_4_ catalyst with GO, which demonstrated an 85% degradation rate for TC and a 99% degradation rate for DOX within 60 min of treatment time. The degradation efficiencies were improved through doping and further enhanced by adding GO and magnetic CoFe_2_O_4_. Numerous ternary compounds (see below in Table 5) that incorporate GO as a co-catalyst have been employed in the photocatalysis field to remove antibiotics. For instance, g-C_3_N_4_/Ag_2_CO_3_/GO [108], BiOBr/MoS_2_/GO [111], and g-C_3_N_4_/GO/CoFe_2_O_4_ [64].

rGO is also considered as a promising semiconductor material, but its characteristics can differ based on the level of reduction and the specific production techniques employed. GO is a type of insulating material that is made from graphene by oxidizing graphite. The reduction process eliminates functional groups in GO that contain oxygen, restoring π-conjugated structures and electrical conductivity. rGO has a uniform tendency to attract molecules, but its ability to react to light is limited because of its wide bandgap and inefficient absorption of light. Therefore, suitable modifications to the base catalyst considerably improve the photocatalysis efficiency [82]. For instance, combining TiO_2_ and rGO makes it more energy-efficient than traditional photocatalysts such as TiO_2_. The enhanced activity of these heterojunctions was determined under simulated solar light; 87% of sulfamethoxazole (SMX) was removed within 1 h [112]. Adding Ag_3_PO_4_ to N-doped rGO yielded higher degradation rates of SMX [113]. Incorporating metal oxides into rGO enhances the photosensitization. The process of degrading different antibiotics in wastewater through photoactive oxidation has been investigated utilizing various graphene oxide-based materials such as rGO/WO_3_, g-C_3_N_4_/BiVO_4_/rGO, BiVO_4_/TiO_2_/rGO, rGO/Bi_4_O_5_Br_2_, rGO-BiVO_4_-ZnO, rGO-ZnO, ferrocene-rGO-ZnO, α-Fe_2_O_3_/ZnO/rGO, CdS-Bi_2_MoO_6_/rGO, and rGO-modified tin selenide (see below in Table 5).

#### Tungsten oxide-based materials or composites

Tungsten trioxide (WO_3_) is considered environmentally benign, making it a preferable option for eco-friendly water treatment applications. In aqueous solutions, WO_3_ is mechanically strong and physiochemically stable, and the synthesis of high-purity WO_3_ is a good option for the degradation of antibiotics under solar light irradiation [114]. WO_3_ is a catalyst that responds to visible light and has consistent physicochemical properties because of its low bandgap energy (2.4 to 2.8 eV) [114]. However, the usage of this material in environmental remediation processes is questionable [115–117] because the electrons generated by light in the CB of WO_3_ (about +0.5 V vs NHE) have a weaker positive potential compared to the reduction potential of O_2_ (O_2_/O_2_^•−^ = −0.33 V vs NHE). Diverse approaches have been suggested to enhance its photocatalytic activity. For example, g-C_3_N_4_-WO_3_ [115], rGO/WO_3_ [116], and multi-wall carbon nanotubes-WO_3_ [118] were found to photodegrade SMX under 300 W xenon arc solar simulator light. These three catalysts showed remarkable degradation of SMX, with degradation rates of 91.7%, 98.0%, and 88.5%, respectively. Various composites were synthesized using a simple anion-exchange approach, including AgI/WO_3_ [119], Ag_3_VO_4_/WO_3_ [117], and AgCl/Ag_3_PO_4_/g-C_3_N_4_ [120]. These composites were prepared by adding different molar ratios of AgX. With the inclusion of AgX, there was a significant enhancement in the removal rate of antibiotics. This was achieved by augmenting the positive potential and accelerating electron transfer rates. The most recent research on using tungsten oxide as photocatalyst to remove antibiotics from water is compiled below in Table 6.

**Table 1:** Degradation of antibiotics by titanium dioxide-based photocatalysts.^a^

Antibiotic	Dosages of antibiotics and catalysts	Light source and other parameters	Removal efficiency (%)	Reaction time (min)	Ref.

clarithromycin	0.1 mg/L of CLA, 100 mg/L of graphene-modified TiO_2_	1000 W xenon lamp, simulated sunlight, pH 6, RWW	86.0	60	[112]
sulfamethoxazole	10 mg/L of SMX, 1250 mg/L of TiO_2_/pBC	50 W xenon lamp, visible light, pH 4 to 10, SWW	91.27	180	[121]
sulfamethoxazole	10 mg/L of SMX, 1250 mg/L of Zn-TiO_2_/pBC	50 W xenon lamp, visible light, pH 4, RWW	81.21	180	[122]
sulfamethoxazole	30 mg/L of SMX, 1000 mg/L of F-Pd co-doped-TiO_2_	320 W xenon lamp, simulated sunlight, UPW	94.2	20	[123]
ciprofloxacin	0.5 mg/L of CIP, 0.43 g/L of black Ti^3+^/N-TiO_2_ (b-N-TiO_2_)	5 W LED, visible light, pH 6.7, UPW	98.5	70	[124]
ciprofloxacin	75 uM of CIP/LEV, 1000 mg/L of N and C co-doped TiO_2_	300 W xenon lamp, visible light, RWW	68.7	120	[63]
levofloxacin			95.7		
tetracycline	10 mg/L of TC/CTC, 300 mg/L of TiO_2_-MOF	300 W xenon lamp, visible light, pH 7, UPW	87.03	60	[125]
chlortetracycline			78.91		
ceftriaxone	100 mg/L of CTR, 1 g/L of activated carbon based TiO_2_	50 W LED, visible light, RWW	99.6	240	[126]
enrofloxacin	10 mg/L of ENF, 1 g/L of MX-TiO_2_ composite	UVA lamp, 2.1 mW/cm^2^, pH 4.8, UPW	91.6	300	[127]
cefixime	20.5 mg/L of CFX, 0.012 g/L of α-Fe_2_O_3_@TiO_2_	500 W halogen lamp, visible light, pH 4.76, SWW	98.8	103	[72]
tetracycline	20 mg/L of TC, 0.01 g/L of Ti^3+^ doped-TiO_2_	500 W halogen lamp, visible light, pH 4.7, SWW	96	60	[75]

^a^RWW: real wastewater, SWW: synthetic wastewater, and UPW: ultrapure water

**Table 2:** Degradation of antibiotics by zinc-based photocatalysts.^a^

Antibiotic	Dosages of antibiotics and catalysts	Light source and other parameters	Removal efficiency (%)	Reaction time (min)	Ref.

erythromycin	10 mg/L of ERY, 50 mg/L of ZnIn_2_S_4_	100 W iodine gallium lamp, visible light, UPW	100	180	[77]
spiramycin	20 mg/L of spiramycin, 1000 mg/L g-C_3_N_4_/ZnFe_2_O_4_	300 W xenon lamp, visible light, UPW	95.0	240	[78]
tetracycline	40 mg/L of TC, 1000 mg/L of Ag_3_PO_4_/Zn-Al LDH	500 W xenon lamp, simulated sunlight, UPW	96	90	[128]
ciprofloxacin	10 mg/L of CIP, 500 mg/L of ZnSnO_3_	350 W xenon lamp, simulated sunlight, pH 5.9, SWW	85.9	100	[129]
ciprofloxacin	10 mg/L of CIP, 1 g/L of Cr_2_O_3_@ZnO	300 W xenon lamp, visible light, pH 3.5, UPW	100	30	[79]
tetracycline	30 mg/L of TC, 1 g/L ZnO and N,O-containing biochar (ZnO/NOC)	350 W xenon lamp, sunlight, pH 6, UPW	97.08	140	[81]
ciprofloxacin	0.05 mg/L of CIP, 2 g/L of MoS_2_/ZnO composites	250 W metal halide lamp, ultraviolet light, UPW	96.18	120	[76]
tetracycline	40 mg/L of TC, 200 mg/L of ZnO/TiO_2_ composites	300 W xenon lamp, simulated sunlight, UPW	84.06	120	[80]

^a^SWW: synthetic wastewater and UPW: ultrapure water

**Table 3:** Degradation of antibiotics by bismuth-based photocatalysts.^a^

Antibiotic	Dosages of antibiotics and catalysts	Light source and other parameters	Removal efficiency (%)	Reaction time (min)	Ref.

tetracycline	20 mg/L of TC, 1 g/L of BiOBr	10 W LED, visible light, pH 6.35, DW	80.3	90	[93]
ofloxacin	10 mg/L of OFX/CIP/NOR, 250 mg/L of BiOCl	125 W mercury lamp, UV light, pH 7, UPW	93.0	80	[83]
ciprofloxacin			74.0	240	
norfloxacin			92.0	240	
tetracycline	20 mg/L of TC, 10 mg/L of CIP/ NOR, 500 mg/L of BiOCl	250 W xenon lamp, visible light, pH 7, UPW	91.90	30	[91]
ciprofloxacin			82.11	120	
norfloxacin			74.52	120	
ciprofloxacin	10 mg/L of CIP, 500 mg/L of BiOCl/NGQDs	300 W xenon lamp, visible light, pH 7, DW	94.0	10	[130]
ciprofloxacin	20 mg/L of CIP, 1000 mg/L of Ti_3_C_2_-Bi/BiOCl	300 W xenon lamp, UPW	89.0	100	[131]
tetracycline	10 mg/L of TC, 667 mg/L BiOCl/CdS	300 W xenon lamp, visible light, UPW	95.9	60	[95]
oxytetracycline	10 mg/L of OTC, 250 mg/L g-C_3_N_4_/BiOCl/CdS	natural sun light, pH 7, DW	99.0	240	[132]
tetracycline	10 mg/L of TC, 200 mg/L Z-scheme BiOCl/Bi–Bi_2_O_3_	300 W xenon lamp, visible light, UPW	94.79	150	[69]
tetracycline	20 mg/L of TC, 400 mg/L BiOCl_0.9_I_0.1_/β-Bi_2_O_3_	350 W xenon lamp, simulated sunlight, pH 6, UPW	82.4	120	[133]
tetracycline	45 mg/L of TC, 200 mg/L MnO_2_/Bi_2_O_3_, 300 mg/L of peroxymonosulfate (PMS)	300 W xenon lamp, visible light, pH 6.5, UPW	73.34	100	[86]
tetracycline	10 mg/L of TC/CTC/OTC/DOX, 1000 mg/L of BiVO_4_/TiO_2_/RGO	1000 W xenon lamp, visible light, UPW	96.2	120	[134]
chlortetracycline			97.5		
oxytetracycline			98.7		
doxycycline			99.6		
tetracycline	190 mg/L of TC, 500 mg/L of BiVO_4_/MoO_3_	300 W xenon lamp, solar light, pH 4.52, UPW	97.66	160	[135]
tetracycline	10 mg/L of TC, 250 mg/L of MnFe_2_O_4_/BiVO_4_	300 W xenon lamp, visible light, UPW	92.0	120	[136]
tetracycline	40 mg/L of TC, 1.5 g/L of BiVO_4_/MoS_2_	100 W xenon lamp, visible light, UPW	97.46	90	[96]
lomefloxacin	25 mg/L of LOM, 500 mg/L of ZnFe_2_O_4_/BiVO_4_/g-C_3_N_4_	300 W xenon lamp, visible light, UPW	96.1	105	[137]
tetracycline	40 mg/L of TC, 10 mg/L of CIP, 1.0 g/L of BiVO_4_@BiOCl	500 W xenon lamp, visible light, UPW	90.32	200	[138]
ciprofloxacin			71.32		
tetracycline	15 mg/L of TC, 500 g/L of Bi_2_WO_6_/CuBi_2_O_4_	300 W xenon lamp, visible light, UPW	91.0	60	[139]
ciprofloxacin	10 mg/L of CIP/ NOR, 1g/L of Mg/Bi_2_WO_6_	300 W xenon lamp, visible light, UPW	99.1	150	[98]
norfloxacin			89.44		
norfloxacin	10 mg/L of NOR, 600 mg/L of Ag_3_PO_4_/Bi_2_WO_6_/MWCNTs	1000 W xenon lamp, visible light, UPW	94.34	180	[140]
ciprofloxacin	10 mg/L of CIP, 500 mg/L of 5 wt % Co_3_O_4_/Bi_2_WO_6_/PMS, 0.2 g/L of PMS	300 W xenon lamp, visible light, pH 6.5, UPW	86.2	5	[141]
ciprofloxacin	10 mg/L of CIP, 5 mg/L of Bi_3_TaO_7_ QDs/g-C_3_N_4_	86 W blue LED, pH 7, UPW	91.0	120	[70]
sulfamethoxazole	5 mg/L of SMX, 500 mg/L of Ag/g-C_3_N_4_/Bi_3_TaO_7_	300 W xenon lamp, visible light, pH 6.5, UPW	98	25	[71]
ciprofloxacin	20 mg/L of CIP, 250 mg/L of CdS-Bi_2_MoO_6_/RGO	500 W xenon lamp, visible light, UPW	91.0	60	[142]
tetracycline	15 mg/L of TET, 600 mg/L of CuInS_2_/Bi_2_MoO_6_	300 W xenon lamp, visible light, pH 7, UPW	84.7	120	[143]
ciprofloxacin	10 mg/L of CIP, 20 mg/L of TC, 250 mg/L of Bi_4_O_5_Br_2_/CdS	350 W xenon lamp, visible light, UPW	85	60	[144]
tetracycline			85	60	
norfloxacin	10 mg/L of NOR, 250 mg/L of ZnO/Bi_2_WO_6_	natural sunlight, pH 6.6, UPW	99.7	120	[145]
levofloxacin	15 mg/L of LEV, 900 mg/L of Bi_2_CrO_6_/g–C_3_N_4_	100 W LED, visible light, pH 6, UPW	92.5	120	[146]
chlortetracycline	20 mg/L of CTC, 900 mg/L of Bi_2_WO_6_/g-C_3_N_4_/ACF	300 W xenon lamp, visible light, SWW	90.2	69	[147]
ciprofloxacin	10 mg/L of CIP, 20 mg/L of TC, 500 mg/L of BiVO_4_/Bi_2_WO_6_/WO_3_	300 W xenon lamp, simulated sunlight, pH 6, UPW	67.5	100	[148]
tetracycline			33.2	75	
tetracycline	20 mg/L of TC/ OTC, 400 mg/L of In_2_O_3_/Bi_2_WO_6_	300 W xenon lamp, simulated sunlight, UPW	86	70	[149]
oxytetracycline			82		
norfloxacin	10 mg/L of NOR/ LEV, 400 mg/L of g-C_3_N_4_/Bi_2_WO_6_	300 W xenon lamp, simulated sunlight, UPW	85.75	120	[150]
levofloxacin			85.82		
chloramphenicol	50 mg/L of CHL, 1 g/L of Ti-Bi_2_WO_6_@BC	500 W xenon lamp, visible light, pH 7, UPW	92.44	120	[97]

^a^SWW: synthetic wastewater, UPW: ultrapure water, and DW: distilled water

**Table 4:** Degradation of antibiotics by graphitic carbon nitride-based photocatalysts.^a^

Antibiotic	Dosages of antibiotics and catalysts	Light source and other parameters	Removal efficiency (%)	Reaction time (min)	Ref.

tetracycline	40 mg/L of TC, 400 mg/L of g-C_3_N_4_	300 W xenon lamp, visible light, pH 4.18, UPW	84.1	90	[101]
tetracycline	20 mg/L of TC, 1 g/L of Nb_2_O_5_/g-C_3_N_4_	250 W xenon lamp, simulated sunlight, pH 3, SWW	90.1	150	[151]
sulfamethazine	10 mg/L of SMT, 101 g/L of C doping g-C_3_N_4_	300 W xenon arc lamp, visible light, UPW	98.0	60	[152]
sulfamethazine	30 mg/L of SMT, 500 mg/L of g-C_3_N_4_	300 W xenon arc lamp, visible light, pH 3, UPW	99.7	60	[153]
erythromycin	50 mg/L of ERY, 500 mg/L of CdS/g-C_3_N_4_	35 W xenon lamp, visible light, pH 5, UPW	81.02	60	[106]
tetracycline	150 mg/L of TC, 400 mg/L of CoO/g-C_3_N_4_	300 W xenon lamp, visible light, UPW	73.12	300	[107]
metronidazole	10 mg/L of MTZ, 1000 mg/L of P-doped g-C_3_N_4_/Co_3_O_4_	250 W xenon lamp, visible light, UPW	70	180	[58]
sulfamethoxazole	5 mg/L of SMX, 1000 mg/L Ag-P-codoped g-C_3_N_4_	350 W xenon lamp, visible light, pH 9, SWW	99	30	[62]
sulfamethazine	5 mg/L of SMT, 200 mg/L 2D/1D g-C_3_N_4_/TNTs	450 W xenon lamp, pH 7, UWW	100	300	[154]
tylosin	25 mg/L of TYL, 500 g/L of Sm-doped gC_3_N_4_	35 W xenon lamp, simulated sunlight, pH 3–11, UPW	78.4	90	[59]
tetracycline	50 mg/L of TC, 11 g/L of ZnO/g-C_3_N_4_	500 W mercury lamp, UV light, RWW	93.4	120	[105]
tetracycline	10 mg/L of TC, 10 mg/L of pyridazine doped g-C_3_N_4_	300 W xenon lamp, visible light, pH 6, UPW	95	60	[103]
pefloxacin	10 mg/L of PEF/ENR/CIP, 50 mg/L of g-C_3_N_4_/TCNQ-X	300 W xenon lamp, visible light, UPW	91.6	180	[102]
tetracycline	8 × 10^−5^ M of TC/DOX, 60 mg/L of K,P-co-doped g-C_3_N_4_/GO/CoFe_2_O_4_	500 W halogen lamp, visible light, pH 7, UPW	85	60	[64]

^a^RWW: real wastewater, SWW: synthetic wastewater, and UPW: ultrapure water

**Table 5:** Degradation of antibiotics by graphene oxide-based photocatalysts.^a^

Antibiotic	Dosages of antibiotics and catalysts	Light source and other parameters	Removal efficiency (%)	Reaction time (min)	Ref.

amoxicillin	50 mg/L of AMX, 400 mg/L of GO/TiO_2_	36 W UV lamp, UV light, pH 6, UPW	99	60	[155]
tetracycline	50 mg/L of TC, 50 mg/L of GO/ZnO	300 W xenon lamp, visible light, pH 11, UPW	74	100	[156]
tetracycline	20 mg/L of TC, 30 mg/L of g-C_3_N_4_/Ag_2_CO_3_/GO	300 W xenon lamp, visible light, UPW	97.6	100	[108]
ciprofloxacin	10 mg/L of CIP, 20 mg/L of BiPO_4_@GO-MMIPs	300 W xenon lamp, visible light, UPW	100	80	[157]
tetracycline	20 mg/L of TC, 25 mg/L of BiOBr/MoS_2_/GO	300 W xenon lamp, visible light, pH 11, UPW	98	40	[111]
trimethoprim	20 mg/L of TMP, 2.5 mg/L of CuFe-LDH/GO	18 W Philips TL-D, UV light, pH 8.8, UPW	90.8	120	[158]
sulfamethoxazole	0.10 mg/L of SMX, 100 mg/L of TiO_2_-rGO	1000 W xenon lamp, simulated sunlight, pH 5.2–6.2, RWW	87.0	60	[112]
sulfamethoxazole	20 mg/L of SMX, 200 mg/L of Ag_3_PO_4_/N-doped rGO	250 W xenon arc lamp, visible light, pH 5.8, UPW	93.8	60	[113]
ciprofloxacin	10 mg/L of CIP, 300 mg/L of rGO-BiVO_4_-ZnO	tungsten lamp (150 mW·cm^−2^), visible light, pH 7, UPW	98.4	60	[159]
ciprofloxacin	10 mg/L of CIP, 20 mg/L of NOR/TC, 50 mg/L of rGO/Bi_4_O_5_Br_2_	500 W of xenon lamp, visible light, UPW	97.6	60	[160]
norfloxacin			80.7		
tetracycline			98.7		
chloramphenicol	1000 mg/L of CAP, 500 mg/L of rGO-ZnO	4 W of UVP compact lamp, UV light, pH 2, UPW	90.8	40	[161]
oxytetracycline	10 mg/L of OTC, 300 mg/L of cobalt ferrite/rGO	300 W xenon lamp, visible light, pH 7.36, UPW	84.7	40	[162]
tetracycline	25 mg/L of TC, 25 mg/L of rGO/MoO_3_/TiO_2_	300 W xenon lamp, visible light, UPW	94	80	[163]
ciprofloxacin	10 mg/L of CIP/SMX, 25 mg/L of Fc@rGO-ZnO	10 W UV lamp, UV light, pH 4, UPW	97.09	180	[82]
ofloxacin	40 mM of OFX, 20 mg/L of rGO-ZnS-CuS	tungsten lamp (300 mW·cm^–2^), visible light, UPW	86.65	135	[164]
oxytetracycline	20 mg/L of OTC, 370 mg/L of rGO/α-Fe_2_O_3_/ZnO, 2.06 mM of PS	24 W LED, visible light, pH 4, UPW	98	90	[110]
norfloxacin	10 mg/L of NOR, 0.75 mg/L of rGO–SnSe	300 W xenon lamp, visible light, pH 7, UPW	90.7	70	[165]

^a^RWW: real wastewater and UPW: ultrapure water

**Table 6:** Degradation of antibiotics by tungsten oxide-based photocatalysts.^a^

Antibiotic	Dosages of antibiotics and catalysts	Light source and other parameters	Removal efficiency (%)	Reaction time (min)	Ref.

sulfanilamide	10 mg/L of SAM, 500 mg/L of Ag doped WO_3_	200 W xenon arc lamp, visible light, pH 5, UPW	96.2	300	[166]
tetracycline	35 mg/L of TC, 1000 mg/L of AgI/WO_3_	300 W xenon lamp, visible light, pH 6.62, UPW	75.0	60	[119]
tetracycline	10 mg/L of TC, 500 mg/L of Ag_3_VO_4_/WO_3_	300 W xenon lamp, visible light, UPW	71.2	30	[117]
sulfamethoxazole	10 mg/L of SMX, 1 g/L of WO_3_/g-C_3_N_4_	300 W xenon lamp, simulated sunlight, UPW	91.7	240	[115]
sulfamethoxazole	10 mg/L of SMX, 2 g/L of WO_3_/rGO	200 W xenon arc lamp, simulated sunlight, pH 7, RWW	98.0	80	[116]
sulfamethoxazole	10 mg/L of SMX, 2 g/L of WO_3_-MWCNTs (WO_3_-CNT)	300 W xenon arc lamp, simulated sunlight, UPW	88.5	180	[118]
tetracycline	50 mg/L of TC, 50 mg/L of Cu-doped WO_3_	300 W xenon lamp, visible light, pH 10, UPW	93.7	60	[167]
levofloxacin	20 mg/L of LVX, 50 mg/L of WO_4_	300 W xenon lamp, visible light, pH 7, UPW	84.3	120	[168]

^a^RWW: real wastewater and UPW: ultrapure water

## Conclusion

This review comprehensively explores the latest advancements and challenges in using semiconductor-based photocatalysts to degrade antibiotic contaminants in the environment. It emphasizes the negative impact of antibiotic discharge on ecosystems and human health. The study investigates the mechanisms involved in photocatalytic degradation, which rely on generating free radicals and reactive oxygen species. Because the use of solar radiation and visible light sources for photocatalytic activation is currently limited, there is a distinct and urgent need for further exploration and development in this field. Therefore, focusing on long-term antibiotic removal rates and conducting comprehensive studies on intermediate compounds is essential.

The present review also extensively covers different strategies to improve the semiconductor photocatalytic activity, including modifying the morphology/structure, constructing heterojunctions, doping metals and nonmetals into the photocatalyst surface, and making surface/interface modifications. These strategies have shown significant improvements in the efficiency of antibiotic degradation by either narrowing the bandgap of the photocatalyst or improving the migration of photogenerated charge carriers and promoting efficient charge separation, contributing to the development of sustainable and highly effective photocatalytic systems. However, despite remarkable results, several challenges need to be addressed, such as the presence of multiple sources of pollutants in wastewater, the complex nature of antibiotics, the necessity to understand the mechanism of photocatalytic degradation at the atomic level, and the development of catalysts that can withstand negative influences from environmental matrices.

To further advance the field of photocatalysis, future research should delve into new and innovative approaches, including developing novel materials, optimizing catalyst preparation methods, enhancing photocatalyst recycling capabilities, and exploring synergistic treatments. The focus should be on designing photocatalysts with quasi-identical photoactivity and ease of separation to minimize waste and reduce operating costs. It is also suggested to perform cost-efficiency analyses, comparing the energy requirements for conventional treatment methods such as AOPs with photocatalysis process for large-scale wastewater treatment.

Extensive research on sustainable and recyclable photocatalysts is urgently needed to tackle environmental concerns related to antibiotic degradation effectively. Strong political support, adequate funding, and effective interdisciplinary collaboration are crucial for advancing progress in this field and successfully applying laboratory findings to real-world solutions. Through careful consideration of these challenges and utilization of cutting-edge technologies, semiconductor-based photocatalytic active nanomaterials offer a promising opportunity to mitigate antibiotic pollution and protect the well-being of our environment.

Applications involving the use of semiconductor-based nanoscale photocatalysts for environmental remediation relate directly to several sustainable development goals (SDGs), specifically SDG 3 (Good Health and Well-Being), SDG 6 (Clean Water and Sanitation), and SDG 12 (Responsible Consumption and Production). Nanoscale photocatalysts contribute to SDG 3 by reducing waterborne diseases and health risks through the effective degradation of toxic pollutants, antibiotics, antibiotic-resistant bacteria, and emerging contaminants in wastewater. Additionally, these advanced materials contribute to SDG 6 by offering environmentally friendly and energy-efficient solutions for water management. Furthermore, their integration into resource-efficient systems supports SDG 12 by minimizing chemical usage and production of waste through a circular approach to environmental management.

## Data Availability

Data sharing is not applicable as no new data was generated or analyzed in this study.
